# 

**Published:** 1916-02-26

**Authors:** 


### CONTENTS.

PAGE

**The Pharmaceutical Society and the Sale of Laudanum** ... ... ... ... ... ... ... 465

**The Future of Medical Education** ... ... 466

**Hospital and Institutional News** ... ... ... 467

The Father in Our King—St. George's Hospital—Sir William Turner—A Canvas-Camp Journal from Malta—The Squire of Hawarden—Assessors in Compensation Cases—Pathological Work at Maudsley Hospital—The Lambeth Medical Boycott—A Case of Leakage—A Government Hospital in Peking—The Liscard Hospital Affair—War Wastage—The Hospital Treatment of Sick Prisoners of War—The Roll of Hospital-Houses—A Hospital Secretary Among the Poets—Public Libraries and Hospital Patients: A New Scheme—A New Proposal for Regulating Street Collections—The Tramway Collections in Manchester—A Useful Committee—The Computation of Superannuation Allowances—The Medical Staff at Kingston Poor-Law Infirmary—Wrapping-Paper for Food—The Indispensable Attendant—Important Alteration in the Poison Schedule—The American Women's War Hospital—A Demand for Hospital Beds for Advanced Consumptives—This Week's Drug Market—Will Every Reader Please Help?

**Training in Surgical Efficiency** ... ... ... 473

An Original College of Surgery.

**The Institutional Treatment of Venereal Disease** ... ... ... ... ... ... 474

Isolation and Other Knotty Problems.

**A Medical Causerie** ... ... ... ... ... ... 475

**Patriotism in British Hospitals** ... ... ... 476

IV. St. Mary's Hospital Medical School.

PAGE

**The Welsh National School of Medicine** ... 477

Colonel Bruce Vaughan's Assured Victory.

**Parliament and the Profession** ... ... ... 478

**The Matrons' and Sisters' Department** ... 479

Is State Recognition in Sight?
Welcome Contributions.
The Dug-out and the Nurse.
Control of Military Nursing.
Services Meriting Distinction.
Liverpool Royal Infirmary.
Cigarette Smoking.

**Female Nurses in the Male Wards of Mental Hospitals in Scotland** ... ... ... ... 481

By George M. Robertson, M.D., F.R.C.P. Edin.
The Hospitalisation of the Asylum.
Objections and Difficulties.

**Rat Extermination in Institutions** ... ... 484

**National Insurance Act Developments** ... ... 485

A Committee of Inquiry Appointed.

**Passing Events and Topics** ... ... ... ... 486

**The Editor's Letter-Box** ... ... ... ... 487

Domestic Economy in the Isolation Hospital.
Medical Papers in Public Libraries.
How to Sell Waste Paper.
The New Wards at Newcastle Union.
Florence Nightingale and the War.
The Uniform System of Accounts.

**Forthcoming Publications** ... ... ... ... 488

**A Fruitful Week** ... ... ... ... ... iv

**Medical Appointments** ... ... ... ... iv

**Buildings Contemplated** ... ... ... ... iv

**The Book Market** ... ... ... ... ... iv

**The Institutional Worker** ... ... (Suppl.) 1

Hospital Fuel and Lighting.

